# Family Planning Knowledge, Attitude and Practice among Married Couples in Jimma Zone, Ethiopia

**DOI:** 10.1371/journal.pone.0061335

**Published:** 2013-04-23

**Authors:** Tizta Tilahun, Gily Coene, Stanley Luchters, Wondwosen Kassahun, Els Leye, Marleen Temmerman, Olivier Degomme

**Affiliations:** 1 College of Public Health and Medical Sciences, Jimma University, Jimma, Ethiopia; 2 Rhea, Research Center on Gender and Diversity, Brussels University, Bussels, Belgium; 3 International Centre for Reproductive Health, Department of Obstetrics and Gynecology, Ghent University, Ghent, Belgium; 4 Burnet Institute, Monash University, Victoria, Australia; Tehran University of Medical Sciences, Iran (Islamic Republic of)

## Abstract

**Background:**

Understanding why people do not use family planning is critical to address unmet needs and to increase contraceptive use. According to the Ethiopian Demographic and Health Survey 2011, most women and men had knowledge on some family planning methods but only about 29% of married women were using contraceptives. 20% women had an unmet need for family planning. We examined knowledge, attitudes and contraceptive practice as well as factors related to contraceptive use in Jimma zone, Ethiopia.

**Methods:**

Data were collected from March to May 2010 among 854 married couples using a multi-stage sampling design. Quantitative data based on semi-structured questionnaires was triangulated with qualitative data collected during focus group discussions. We compared proportions and performed logistic regression analysis.

**Result:**

The concept of family planning was well known in the studied population. Sex-stratified analysis showed pills and injectables were commonly known by both sexes, while long-term contraceptive methods were better known by women, and traditional methods as well as emergency contraception by men. Formal education was the most important factor associated with better knowledge about contraceptive methods (aOR = 2.07, p<0.001), in particular among women (aOR_women_ = 2.77 vs. aOR_men_ = 1.49; p<0.001). In general only 4 out of 811 men ever used contraception, while 64% and 43% females ever used and were currently using contraception respectively.

**Conclusion:**

The high knowledge on contraceptives did not match with the high contraceptive practice in the study area. The study demonstrates that mere physical access (proximity to clinics for family planning) and awareness of contraceptives are not sufficient to ensure that contraceptive needs are met. Thus, projects aiming at increasing contraceptive use should contemplate and establish better counseling about contraceptive side effects and method switch. Furthermore in all family planning activities both wives' and husbands' participation should be considered.

## Background

The lifetime risk of maternal mortality of women in sub-Saharan Africa is 1 in 39 live births, which is the highest when compared to other world regions. The World Health Organization (WHO) estimated in 2012 that 287,000 maternal deaths occurred in 2010; sub-Sahara Africa (56%) and Southern Asia (29%) accounted for the global burden of maternal deaths [1]. One of the targets of the Ethiopian Ministry of Health, with respect to improving maternal and child health, is to increase the contraceptive prevalence rate (CPR) from 32% to 66% by 2015. In order to achieve this target, the Ministry has given priority to the provision of family planning services in the community [2].

With 87 million people, Ethiopia is the second most populous nation in sub-Saharan Africa, with a continuing fast growing population of 2.7% per year [3]. The maternal mortality ratio (MMR) is 676 per 100 000 women aged 15 to 49, with an estimated 32% of all maternal deaths attributed to unsafe abortions [4]. A study conducted in Northwest Ethiopia in 2005 indicated that prevalence rates of spontaneous and induced abortions were estimated at 14.3% and 4.8% of all pregnancies respectively [5].

Despite the recent increase in contraceptive use, sub-Saharan Africa is still characterized by high levels of fertility and a considerable unmet need for contraception [6]. The total fertility rate in Ethiopia is 4.8 births per woman and is considerably higher in the rural then the urban areas. Observed fertility rates among women are 33% higher than the wanted fertility rates. In absolute numbers, this means 0.6 additional children in urban areas and 1.5 in rural areas. This is particularly the case in Oromiya region where the total fertility rate is as high as 5.6 children per woman and 30% of the currently married women have an unmet need for contraception which represents the highest figure of all regions in Ethiopia [4]. In the five years preceding the Ethiopia demographic Health Survey (EDHS) in 2011 it is estimated that, three births of every four (72%) were wanted at that time, 20% were wanted but not at the time of pregnancy, and 9% were unwanted [4]. A better use of family planning could reduce many of these mistimed and unplanned pregnancies, while at the same time it could reduce the number of unsafe abortions as well as the mortality related with child birth [7].

On the other hand, couples have a right to choose and decide upon the number of children they desire. This means that both partners have the right to be involved in fertility matters and as such husbands play a crucial role in fertility decision-making in most of the world [8]. Clearly, male-involvement in family planning has positively affected contraceptive use and has caused an overall decline in fertility in the developing world. Men's fertility preferences and attitudes towards family planning seem to influence their wives attitudes towards the use of modern contraceptives [9]. Therefore, programs that attempt to promote reproductive health through increasing the use of modern contraceptives need to target men specifically at all levels of the program. Hence, men should be actively involved at the ‘knowledge’ level (the concept of family planning), the ‘supportive’ level (being supportive for other to use contraception) and the ‘acceptor’ level (as contraceptive user). Their decision-making role should be taken into account in order to promote contraceptive use [10]. Similar research indicates that women's feelings about their partners and about involving men in contraceptive and reproductive decisions must always be taken into account [9]. Previous studies indicated that acceptance of children as God's will, attitudes towards preventing pregnancy, knowledge on different method choice and the understanding of the side effects of different methods are among the factors related to contraceptive use [11], [12]. Moreover, studies on perception of spousal approval and opposition from husbands are positively associated with low contraceptive use [13].

Given the above factors associated with contraceptive use, the primary objective of this study was to examine the contraceptive prevalence rate among married couples and to study the factors that influence contraceptive use. A secondary objective was to determine knowledge on contraceptives (method-specific; including barrier, hormonal, permanent and dual protection methods), and attitudes towards family planning. Finally, fertility preference among married couples was assessed to see the variation between men and women.

## Materials and Methods

This analysis forms part of a baseline assessment for a broader study aimed at determining the effect of a family planning education intervention on the knowledge, attitude and practice of married couples regarding family planning as well as male involvement (will be disseminated separately). The study is conducted in Jimma Zone, one of 14 administrative zones of Oromyia region located in the Southwest of Ethiopia. Its capital, Jimma, is found 352 km to the south west of the national capital, Addis Ababa. Jimma Zone is an area of 15,568.58 Km^2^ with 17 *woredas* (districts) and one special zone. According to the 2007 national census, the total population is 2,486,155, of whom 1,250,527 are men and 1,235,628 women [14].The rural part counts for 89.5% of the total population size of the zone in which the dominant ethnic group is the Oromo. The study area is thus a typical rural setting.

The study population consisted of couples (women and their husbands) who were legally married, lived for more than six months in the study area and of which the wives were 15–49 years (the reproductive age group) but not pregnant at the time of the survey. Husbands within a polygamous marriage (who had more than one wife) were excluded from the analysis to decrease redundancy of information. A multi-stage sampling design was used with districts (*woredas*) as primary sampling units (PSU), and sub-districts (*kebeles*) as secondary sampling units (SSU). The study covered three *woredas* i.e. Seka, Manna and Gomma, in which six *kebeles* were randomly selected: Goyoo qechema, Koffie, Gobiemuleta, Haro, Gembie and Bulbulo. In each selected *kebele*, a complete census of married couples was prepared to use as a sampling frame. Married couples were then randomly sampled from each locality, based on a computer generated random number list until the required size was achieved.

The sample size was computed using Minitab version 14 statistical software in the context of the broader intervention study. Adding 10 percent for non- responses resulted in a final sample size of 427 couples per group or 854 for the entire sample to be drawn equally from each sub-districts.

This study consisted of two parts, including quantitative and qualitative data collection techniques. Data for the quantitative study were collected using semi-structured questionnaires. Separate questionnaires were administered for male and female respondents but with similar contents including socio-demographic characteristics (age, sex, ethnicity, occupational status, income, age at first marriage), reproductive characteristics (number of children, sex preference of couples), as well as question modules on knowledge, attitudes and practice regarding contraceptive use (types of contraception, use of contraception, user perspective, attitudes of a husband and wife towards contraceptives, husband-wife communication on family planning, ever use of contraceptives, current use of contraceptives and reasons for not using contraceptives).

The questionnaire includes not only types of contraceptive as knowledge part but also how to use, where to get family planning service, side effects of contraception and other points too. The survey instruments were developed from a validated questionnaire and were considered valid and reliable through the favorable comments of experts for obtaining information on couples about knowledge, attitude and contraceptive practice [8], [9], [15], [16]. Pilot testing of 5% of the sample revealed that respondents were able to understand and answer questions. Six male and six female data collectors participated in the study and were supervised by three field coordinators. Data collectors were recruited from the local community. We paired the data collectors by sex: men to husbands and female to wives because of the sensitivity of the issue. Interview conducted in private location, each couple at a time but separately keeping the interviewee privacy. Interview conducted if both spouses willing to participate.

For the qualitative data, focus group discussions, using a semi-structured topic guide were employed. Focus group discussions were done to probe to understand the phenomena of couples contraceptive practices within the society. The semi-structured topic guide covered the socio-cultural factors related with contraception and husband's responsibility towards contraception. Four groups consisted of married women and four groups consisted of married men, making a total of eight focus group discussions. Each focus group discussion consisted of 8 to 12 participants. Participants were selected purposively based on who can give the most and best information about coupes contraceptive practice. The participants were married individuals. The group discussions were moderated by university graduates who speak the local language. Similar to the quantitative part, focus group discussions were done female to female and male to male moderators. For the qualitative data participants were first given number a code and their characteristics registered (age and sex). At each time the participant wanted to give an idea first he/she has to call the number. Notes on points of discussion was taken in addition to tape recording.

### Data analysis

The data set for this analysis contained data from 854 husbands and their wives. For the quantitative data analysis, STATA® 10 for Windows® was employed. Analyses were done at the level of the individual independently from the spouses. Simple descriptive analysis was done to explore levels of awareness, knowledge (on different types of contraceptive and knowledge level), attitude and practice among respondents. Bivariate analysis was used to investigate the effect of demographic and socioeconomic variables on fertility preferences and contraceptive practice. Finally, multivariate logistic regression was used to identify predictors of these outcome variables. Statistical significance was considered at p-values less than 0.05.

Qualitative data from focus group discussions were recorded as sound files using tape and subsequently transcribed to text files. Transcripts of the recorded discussions were coded and analysed using thematic areas manually and participants' identifying details were removed. No computer software was used for qualitative data analysis.To check the internal consistency and reliability, data from the quantitative part was used to triangulate with the qualitative results

### Ethical considerations

Ethical clearance of the study was obtained from the research and ethics committee of the College of Public Health and Medical Sciences, Jimma University, Southwest Ethiopia and Ghent University's Ethical Committee in Belgium. Written consent was obtained from each man and woman participating in the study after the data collectors explained about the purpose of the study using a predefined information sheet. Written informed consent was taken from spouses on the behalf of those wives for who were in the age less than 18 years. No compensation was rendered as direct incentive to the participants. The ethics committees approved this consent procedure.

## Results

### Socio Demographic Characteristics

A total of 811 out of 854 sampled couples responded, equating to a response rate of 94.9%. All women were between 15 and 49 years (as per inclusion criteria), with a median age of 30 (IQR = [25;35]). Median age among males was 36 (IQR = [30;45]) (see Table 1).

**Table 1:** Socio-demographic characteristics and reproductive history.

Characteristics at individual level	Male (N = 811)	Female (N = 811)
**Median age**	36 [IQR = 30;45]	30 [IQR = 25;35]
**Education**		
No education	184 (23%)	532 (65%)
Read and write	126 (15%)	54 (7%)
Primary	437 (54%)	204 (25%)
Secondary and above	64(8%)	21 (3%)
**Religion**		
Muslim	737 (91%)	743 (91%)
Orthodox	53(6%)	55 (7%)
Others*	24 (3%)	13 (2%)
**Occupation**		
Farmer	732 (90%)	668(82%)
Merchant	24(3%)	41(5%)
Daily laborer	27(4%)	26(3%)
Others	28 (3%)	76(10)**
**Median age at first marriage**	21[IQR = 20;25]	16[IQR = 15;18]
**Median number of desired children before using contraceptive**	4 [IQR = 3;5]	4[IQR = 3;5]
**Ever had a child who died in childhood** §	NA	296(36.5%)

Characteristics at household level	Households (N = 811)
**Median income of couples**	225 Birr [IQR = 150;370] per month (≈9.3 €)
**Median household size**	5[IQR = 4;6]
**Median number of children alive**	3[IQR = 3;3]
**Median duration of couple's marriage (Years)**	11[IQR = 6;19]

Percent distribution of currently married women and men by their socio demographic characteristics, Jimma zone, Ethiopia 2013.

* Protestant and traditional religions.

§ Data only from wives.

** Majority were housewives.

Almost two-thirds of the women (n = 532, 66%) had not received education, but 204 (25%) had completed primary education; among men, 184 (23%) had not received education while 437 (54%) had completed primary schooling.

Oromos were the principal ethnic group accounting for 1,417 (87%) individuals; 97 (6%) others were Dawro, 28 (2%) Keffa, 25 (2%) Yem, 23 (1%) Amhara, and 32 (2%) from other ethnic groups. The majority of the respondents, 743 (92%) women and 737 (91%) men, were Muslim; second most prevalent religion was Orthodox Christianity with 55 (7%) women and 53 (7%) men. Education levels were different across these two most prevalent religions: 66 out of 1480 (4%) Muslims had completed secondary education in comparison to 16 of the 108 (15%) Orthodox Christians (χ^2^(1, N = 1588) = 19.98, p<0.001). Similarly, Amhara, Yem and Tigrie, of which approximately 25% are Orthodox Christians, showed higher levels of literacy than the other population groups (χ^2^(1, N = 1622) = 10.46, p = 0.001).

Agriculture was the main occupation of the interviewees with 732 (90%) men and 668 (82%) women; 71 (9%) of the women reported being housewives. The median income of couples was 225 Birr (IQR = [150;370]), which is approximately 9.3 Euro, per month, according to information obtained from the wives. Daily laborers had a median income of 150 Birr per month, government employees 700 Birr.

The median household size was 5 (IQR = [4;6]), with a median of 3 children, and 422 (52%) households comprised of five to seven members. Literate respondents had smaller household sizes than the illiterate (χ^2^(11, N = 811) = 28.23, p = 0.003), as well as less children (χ^2^(10, N = 811) = 30.48, p<0.001). The median age at first marriage for men aged 20–59 was 21 (IQR = [20;25]) and 16 (IQR = [15;18) for women aged 20–49. There were 40 (5%) males and 518 (65%) females who married before age 18. The median duration of the couple's marriage was 11 years (IQR = [6;19]). Among the husbands, 209 (26%) stated having been married already prior to the current union.

One-third of the female respondents (n = 296, 36%) reported having ever lost at least one child; 209 (70.6%) reported ever having lost at least one boy, 181 (61%) at least one girl.

More than 98% of the study participants had access to health facilities providing family planning services in their surrounding (at least health post i.e Primary level health care in Ethiopia (can serve 3,000–5,000 individuals).

### Fertility preferences

A majority wanted to have more children: 494 (72%) among the men, 439 (64%) among the women. The median desired number of children before using family planning among both women and men was 4 (IQR = [3;5]). Of the 233 women who had reached or exceeded their desired number of children, 90 (39%) still reported a need for more children; on the other hand, among the men having reached or exceeded that number, 131 out of 252 (52%) wanted more children.

Overall, 413 (44%) respondents of the 933 desiring more children expressed a sex preference for the next child. Among men, 172 (35%) wanted a boy versus 47 (10%) a girl; among women these numbers were respectively 120 (27%) and 74 (17%). Sex preference varied depending on the number of boys and girls already living in the family (see Table 2). Respondents with no boys had a distinct desire to have a boy as the next child. This preference disappeared among women once they had at least one boy and among men once they had two boys. A similar preference for a girl is noticed for respondents that did not have girls yet, although the extent of this preference is more limited. On average, both men and women had a preference for a boy if they had at least one girl.

**Table 2:** Fertility preference.

	Men			Women		
	Boy	Girl	No preference	Boy	Girl	No preference
Number of girls from all marriages						
0	13 (14%)	25 (28%)	52 (58%)	12 (12%)	39 (37%)	53 (51%)
1	55 (37%)	13 (9%)	79 (54%)	39 (26%)	24 (16%)	86 (58%)
2 or more	93 (44%)	4(2%)	114 (54%)	65 (41%)	6 (4%)	89 (55%)
Number of boys from all marriages						
0	56 (64%)	1 (1%)	30 (35%)	51 (49%)	1 (1%)	53 (50%)
1	64 (40%)	7(4%)	91 (56%)	37 (25%)	31 (21%)	80 (54%)
2 or more	40 (20%)	34 (17%)	127 (63%)	26(16%)	38 (24%)	95 (60%)

Percent distribution of Sex preference for next child depending on the number of boys and girls from all marriages living in the family of respondents in Jimma zone, Ethiopia 2013.

### Knowledge about Family Planning

The concept of family planning was well known to respondents: 760 (94%) women and 795 (98%) men responded ever having heard of it. The median number of methods of contraception that were known among men was 5 (IQR = [2;8]) which was the same among women 5 (IQR = [3;6]); the mean was 5.4 for both sexes (95%CI_men_ = [5.2;5.7] and 95%CI_women_ = [5.2;5.5]). As such, there was no statistical difference between the sexes (p = 0.6585). Different levels of knowledge were found across the *kebeles*: only 3 of the 265 (1%) respondents in Haro knew more than 5 methods of contraception compared to values ranging from 34% to 60% for the other *kebeles*. No relationship was found between knowledge level and age, religion or ethnic affiliation. Formal education on the other hand, was associated to a higher knowledgeability about contraceptive methods (aOR = 2.07, p<0.001), in particular among women (aOR_women_ = 2.77 vs. aOR_men_ = 1.49; p<0.01).

Method-specific knowledge levels varied from 12% for vaginal contraceptives (diaphragm, foam, jelly) to 94% for injectable contraceptives. Differences were found between men and women (Table 3). Only short-term hormonal methods like the contraceptive pill and injectable contraceptives were consistently well known by both sexes. Least known were the permanent methods, traditional methods and emergency contraception. Major differences between women and men were noted for the long-term hormonal methods (χ^2^(1, N = 1622) = 217.96, p<0.001) and emergency contraception (χ^2^(1, N = 1622) = 140.12, p<0.001). A total of 1064 (68%) respondents knew how to use contraceptives, with more women (77%) being knowledgeable about it than men (58%) (χ^2^(1, N = 1622) = 67.42, p<0.001). Similarly, knowledge on contraceptive use decreased with increasing age even when correcting for sex (aOR_per additional year of life_ = 0.98; p = 0.003).

**Table 3:** Knowledge of contraceptive methods.

	Total(N = 1622)	Female(N = 811)	Male(N = 811)	P-value
Short-term hormonal methods	1545 (95%)	801 (99%)	744 (92%)	
*Pill*	1482 (91%)	757 (93%)	725 (89%)	0.005
*Injectables*	1517 (94%)	796 (98%)	721 (89%)	<0.0001
Long-term hormonal methods	1297 (80%)	768 (95%)	529 (65%)	
*IUCD*	661 (41%)	419 (52%)	242 (30%)	<0.0001
*Implants/Norplant*	1275 (79%)	754 (93%)	521 (64%)	<0.0001
Barrier methods	1001 (62%)	490 (60%)	511 (63%)	
*Condom*	997 (61%)	488 (60%)	509 (63%)	0.284
*Diaphragm/foam/jelly*	194 (12%)	147 (18%)	47 (6%)	<0.0001
Permanent methods	692 (43%)	296 (36%)	396 (49%)	
*Female sterilization*	665 (41%)	284 (35%)	381 (47%)	<0.0001
*Male sterilization*	205 (13%)	75 (9%)	130 (16%)	<0.0001
Traditional methods	663 (41%)	242 (30%)	421 (52%)	
*Standard days method*	515 (32%)	188 (23%)	327 (40%)	<0.0001
*Lactational Amenorrhea Method*	250 (15%)	74 (9%)	176 (22%)	<0.0001
*Rhythm method*	412 (25%)	175 (22%)	237 (29%)	<0.0001
*Withdrawal*	339 (21%)	158 (19%)	181 (22%)	0.160
Emergency contraception	246 (15%)	37 (5%)	209 (26%)	<0.0001

Percentage of currently married women and men, who have heard of any contraceptive methods, Jimma zone, Ethiopia 2013.

^**^p<0.05 considered as an association.

### Attitudes towards Family Planning

Of the 1622 respondents, 91% (1479) were in favour of family planning; logistic regression showed that factors associated with a more positive attitude towards family planning were: being a man (aOR = 1.67; p = 0.021), young age (aOR_per additional year of life_ = 0.97; p<0.001) and being literate (aOR = 1.89; p = 0.002). Male respondents were asked specifically whether they would support their wives to use family planning. Of the 811 male respondents, 751 (93%) answered positively and 22 (3%) negatively. This finding was corroborated during the focus group discussions with married men.

*“Couples should limit their number of kids for the seek of child's health and for the household economy.” (Male, 18 years)*

### Contraceptive Practice

We did not consider husbands' number of children at first contraceptive use as only 4 (0.2%) males reported having ever used contraceptives. Condom use was thus very low. Among women, 517 (64%) ever used a method of contraception; 350 (43%) were using contraception at the time of the survey. This difference in contraceptive use between men and women was corroborated by the focus group discussions as these showed that both married women and men mostly considered contraceptive use as a woman's task:

*“What will I do in a family planning clinic, contraception is women's business, I will just give my wife the necessary financial support she needs” (Male, 45 years)*

Two hundred sixty five (51%) wives had one to two children at their first contraceptive use. The median number of children a woman had when starting contraception was 2 (IQR = [2-2]) which corresponds to 2 children less than what they considered the ideal number of children.

The most commonly used methods when starting contraception were injectable (316 out of 515, 39%) and oral (174 out of 515, 21%) hormonal contraceptives. The prevalence of these hormonal contraception methods was much related to the age of the woman. Injectable methods were most common among younger women (aOR_per additional year of life_ = 0.94; p<0.001), while oral contraceptives were more frequently used by older women (aOR_per additional year of life_ = 1.06; p<0.001). Of the 350 women who were using contraceptives at the time of the survey, 283(81%) were using injectables and 33 (9%) oral contraceptives.

Multivariate analysis showed that higher current use of contraception among women was associated with being literate (aOR = 1.58; p = 0.005), the number of children (aOR_per additional child_ = 1.11; p = 0.027) and being highly supportive of family planning (aOR = 4.01; p<0.001). Household income didn't show an association with current contraceptive use (p = 0.593). The same factors were also determinants for contraceptives having ever used.

**Figure 1:**
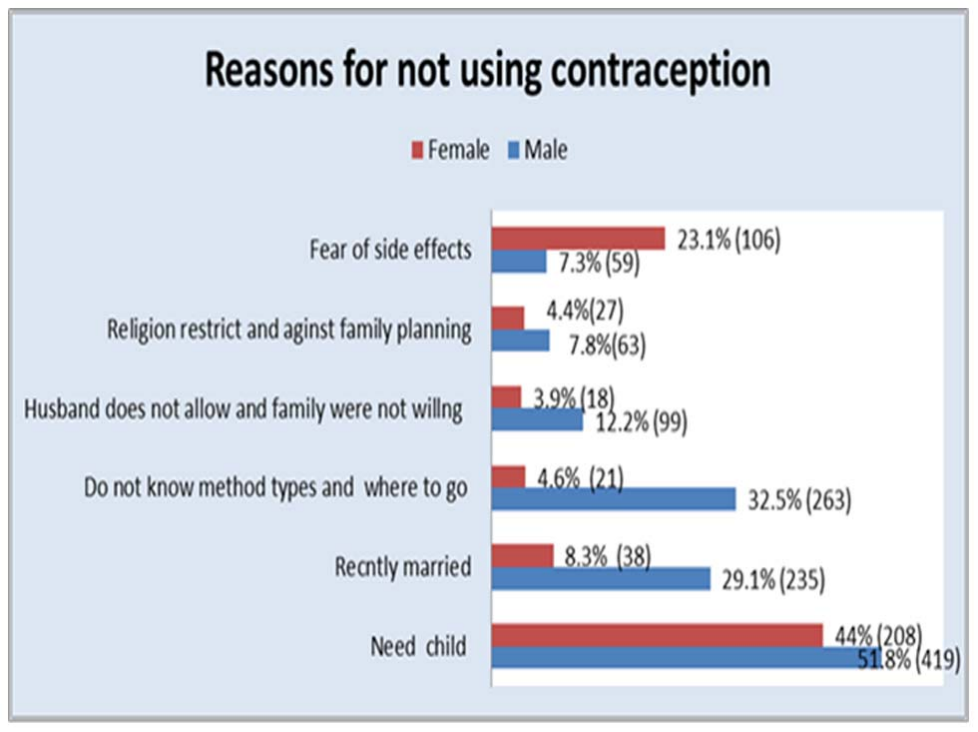
Reasons given for not using contraception among married couples by sex, Jmma zone, Ethiopia 2013.

Reasons given by males for not using contraception included being recently married 235 (29%) and lack of knowledge of the different types of methods 235 (30%). The reason for not using contraception given by both male and females was the desire to have children (419 (51.8%) men and 203 (44%) women). Among women fear of side effects was reported by 106 (23%) as the reason for not using contraception (see Figure 1). Likewise, the qualitative findings also indicate fear of contraceptives' side effects as a barrier to use contraception by women:

*“Women don't use contraceptive because they don't want to get pain by the side effect of pills and injectable” (Female, 25 years)*

Additional results from focus group discussions indicate that males are at least partly responsible for women not using contraceptives:

*“sometimes husbands oppose wife use of contraceptive because they think she does not want to give birth and instead she has an intention to go for another man” (Female, 33 years)*

Among women, 183 (36%) of current contraceptive users reported ever having switched between methods, with 175 (96%) of them giving lack of comfort as one of the reason and 99 (54%) fear of side effects. Likewise the qualitative part supports this result.

*“I used one type of contraceptive and it result in burning sensation and excessive menses so I changed to other contraception method” (Female, 20 years)*

## Discussion

Despite the recent increase in contraceptive use, Ethiopia, Africa's second most populous country, is known to have a low contraceptive prevalence and high total fertility. The objective of the study presented in this paper was to investigate differences among males and females regarding knowledge on contraceptive methods, fertility preference and contraceptive practice among married men and women in Jimma zone, Ethiopia.

The results of this analysis demonstrate that more than 98% of the couples had access to health facilities that deliver family planning. The median household size of five in the study area (Jimma zone) was comparable to the national household size (4.6 persons), especially that of rural areas (4.9 persons) [4]. Literacy was found to be linked to smaller household sizes, which is in line with previous findings [4], [16].

Age at first marriage was lower in our study population compared to national figures. For females aged 20–49 years, the median age at first marriage was 16 years, i.e. one year younger than the national median (17.1 years) and a previously published study from Butajira (16.9 years). Among men aged 20–59, a one year difference with the national median was observed (22 years vs 23.1 years). This also corroborates general trends that men marry at older ages than women [4], [17].

Similar to EDHS (2011), this study revealed that more men than women have a desire for more children [4]. This suggests that the low use of contraception among men is partly a well-reasoned decision, and not only a consequence of limited knowledge. In this study the mean ideal number of children was 4.2 and 4.6 among men and women respectively. This is in contrast to the national figures that show a difference in the mean ideal number of children between men and women, i.e. 5.9 and 4.9 respectively [4].

A study conducted in Tigray, Ethiopia reported that the mean desired number of children among men differed significantly as compared to that of women ( Δ = 1.2; 95%CI: [0.87;1.53]) [10]. The inconsistency with our study could partly be explained by a different formulation of questions since we inquired about the ideal number of children before starting contraception, instead of the actual desired number of children. Furthermore, we identified discrepant results with respect to the desired number of children and the desire to have additional children. Considerable numbers of couples that had reached the desired number of children still desired more. Research should be done exploring the causes of this finding.

With regards to sex preference, respondents with no boys had a distinct desire to have a boy as a next child; the same pattern of wanting a girl was observed among couples that didn't have a girl yet. However, the extent of the preference for a girl was more limited. In addition, the preference was stronger among men, a finding that is supported by the results of a study conducted in Ethiopia in which most men (48%) reported that they would like more sons than daughters [18]. This might be due to cultural norms around son preference or, as suggested by others, the interest for more sons could be based on subsistence reasons, such as economic security and maintaining their status within the traditional family structure [19]. From the focus group discussants, a woman (25 years) described that she wants to have five male and three female; because male stay with me but after marriage female follows her husband. Moreover this study reveals nearly 36% women reported ever had child death of which almost 70% boy child. This could be the other possible expatiation for boy sex preference.

The high level of knowledge on at least one form of contraception among the participants of this study (96%) is in line with previously reported national figures (98.4%). In our study, we observed no significant difference between men and women with regards to knowledge: the average number of methods known in both sexes was 5.4 contraceptive types. In contrast, at national level, the average number of contraceptive methods known by men is higher than women (6.3 and 5.4 respectively) [4]. As such, men included in our study were less knowledgeable about different methods compared to the average Ethiopian man.

In the present study, short-term hormonal contraceptive methods like the pill and injectable contraceptives were consistently well-known by both sexes. Permanent methods, traditional methods and emergency contraception on the other hand were the least known contraceptive methods. Compared to the results from the Ethiopian Demographic Health Survey (2011) women and men are more familiar with long term and standard days methods, but in the case of barrier methods (diaphragm/jelly and male condom) and emergency contraception the reverse is true for the study population [4]. In addition our study identified major differences in knowledge of emergency contraception between the two sexes. The limited knowledge of women on emergency contraception suggests that this type of contraception is not part of the standard information package that is given to women in our study area.

Overall, our respondents had a positive attitude towards family planning (91%), but less than 1% of the males and 64% of the women reported having ever used any type of contraception. Other studies have already described similar findings, i.e high awareness but low utilization of contraceptives, making this situation a serious challenge in developing countries [8], [20]. The EDHS 2011 reported a current contraceptive prevalence rate of 29% for married women, which is lower than our finding (43%) [4]. A reason for this could be that the majority of our respondents have access to health facilities in the study site. With respect to the method-specific contraception, injectables (39%) and oral hormonal contraceptives (21%) were the main methods used. Compared to EDHS 2011, a noteworthy finding in our study is the low use of implants, suggesting that health facilities in our study area are not able to deliver this service.

Among background characteristics of women, literacy, age, the number of children, and being highly supportive of family planning were found to be important indicators of current contraceptive; this is confirmed by different studies [4], [20]–[22]. Fear of side effects was identified as the reason for not using contraceptives among married women, a finding that has been described already in other studies conducted in Ethiopia and Bangladesh [23], [24].

Our qualitative study findings also assured that fear of side effects is one of the most important reasons of not using contraceptives by women. In addition, this study reported that men's reasons for not using contraception were being recently married and the desire for more children. The latter is also one of the most important reasons of not using contraception among women. In general, in the study area the findings indicate a prevailing belief that contraception is only a women's business.

This study has limitations resulting from the design that was used, in the sense that cross-sectional studies do not allow to establish cause-effect relationships. In addition, an important limitation is the exclusion of couples with pregnant women from this baseline study as per the intervention protocol. This clearly affects the contraceptive prevalence rate and could potentially affect some other indicators too. The group of pregnant couples however represented only 7% of all couples from our sampling frame. This leads us to believe that the effect on the figures is probably relatively small. A final potential limitation is reporting bias. It also suffered from social desirability as it is a community based study. In that context, we decided to exclude one *kebele* (Gobbie Mulata) from the analyses of the ideal number of children as there was evidence of an erroneous comprehension of the question.

## Conclusion and Recommendations

The analysis of this study provides information on married men and women on knowledge, attitudes and contraceptive practice in Jimma zone, Ethiopia. Our results demonstrate that good knowledge among males and females was observed, yet differences on knowledge of specific contraception methods exist. The study reveals that mere physical access (proximity to clinics for family planning) and awareness of contraceptives are not sufficient to ensure that contraceptive needs are met. We also noticed the existence of a sex preference for boys both among men and women. Condom use by men is above the national average but it is low compared to most Sub-Saharan African countries. It is evident from this study that high knowledge on contraception is not matched with the high contraceptive use. Among reasons for not using contraception, want to have a child and side effects of contraceptive were given by men and women respectively. Therefore, family planning interventions should pay particular attention to both wives' and husbands' participation in family planning, while at the same time further educating married women and men on specific methods of contraception and their possible side effects. Moreover, a considerable amount of child death mainly boy child linking with boy sex preference reflects family planning interventions to see the ways beyond only for contraceptive purpose.
